# Genetic heterogeneity of hepatitis C virus cell entry receptors seems to have no influence on selection of virus variants

**DOI:** 10.1186/1743-422X-11-50

**Published:** 2014-03-14

**Authors:** Maren Lipskoch, Manfred Wiese, Joerg Timm, Michael Roggendorf, Sergei Viazov

**Affiliations:** 1 Institute of Virology, Essen University Hospital, University of Duisburg-Essen, Essen, Germany; 2 Universitätsklinikum Leipzig, Klinik für Gastroenterologie und Rheumatologie, Sektion Hepatologie, Liebigstr. 20, 04103 Leipzig, Germany

**Keywords:** HCV AD78, HCV cell receptor polymorphism, LDLR, OCLN, SCARB1

## Abstract

**Background:**

No information is available on the possible influence of the genetic heterogeneity of major hepatitis C virus (HCV) cell receptors on selection of virus variants.

**Findings:**

Anti-D globulin preparations contaminated with the HCV strain AD78 caused hepatitis C infection in more than 3000 women in East Germany in 1978. Analysis of the core to NS2 gene sequences of this strain in several globulin batches revealed the presence of three closely related but distinct virus variants of the same strain. Apparently even distribution of these three virus variants was observed in 91 patients infected with the AD78 strain. None of these patients was infected with more than one virus variant, suggesting a selection mechanism of a particular virus variant in each patient. To verify the hypothesis that heterogeneity of HCV cell receptors might influence the virus variant selection, single-nucleotide polymorphisms (SNPs) in low-density lipoprotein receptor (LDLR), occludin (OCLN), and scavenger receptor B1 (SCARB1) genes in AD patients were analyzed. No evident correlation between receptor polymorphisms and presence of a particular virus variant was noted.

**Conclusion:**

SNPs of HCV cell entry receptors have no influence on virus selection in patients infected with an inoculum containing different virus variants.

## 

Accumulating data show that mixed infection with two or several HCV genotypes or subtypes usually is a very low event even among multiple exposed individuals such as intravenous drug users [1]. These data suggest the existence of mechanism(s) that restricts either the coinfection of hepatocytes with two or several virus variants or superinfection or both. Recently, a phenomenon of HCV superinfection exclusion by already infected Huh7 hepatoma cells due to interference at the level of HCV RNA translation and/or replication has been described [2,3]. Such a restriction may also function at other stages of the HCV replicative cycle, including the entry step. Thus, the Huh7 cells infected with one HCV strain demonstrate downregulation of expression of two key factors for HCV entry – claudine-1 and occludin [4].

In favour of the hypothesis that the mechanism(s) of HCV coinfection/superinfection restriction might be operative at the stage of virus entry are data obtained by our group that has initiated a study of a single-source outbreak of HCV infection in about 3000 women in East Germany in 1978 caused by contaminated anti-D globulin [5]. Analysis of the viral core-NS2 and NS3 gene sequences from several anti-D globulin batches revealed that three closely related but still distinct variants of the same HCV AD78 strain were present in all preparations [6]; Viazov S, in preparation. The progeny of the same three virus variants were present in 91 HCV AD78-chronically infected patients (Figure 1). Importantly, none of these anti-D patients harboured more than one variant of the HCV AD78 despite the fact that the inoculum, the anti-D globulin batches, contained all three variants of the virus. These data allowed us to speculate that one or several of the virus-binding receptors in cells from HCV infected patients are heterogeneous and that polymorphism of these receptors might have an influence on HCV variant selection in case of an inoculum containing a mixture of viruses. The anti-D cohort represented a unique group to verify this hypothesis. Therefore, the single-nucleotide polymorphisms (SNPs) in major HCV cell receptor genes [7,8] from anti-D patients were investigated. The SNPs that were associated with nonsynonymous substitutions and, which occurred with unknown frequencies, were chosen for the analysis. According to these criteria, seven SNPs, two for the low-density lipoprotein receptor (LDLR), three for the scavenger receptor B1 (SCARB1), and two for the occludin (OCLN) genes were chosen for subsequent analysis (Table 1). Two of the receptors molecules, CD81 and Claudin-1 were found to be quite conserved.

**Table 1:** The SNPs in the major HCV cell receptor genes chosen for analysis

**Receptor gene**	**Polymorphism**
LDLR	rs11669576
	rs45508991
SCARB1	rs5890
	rs74830677
	rs77554031
OCLN	rs2666626
	rs79095497

**Figure 1:**
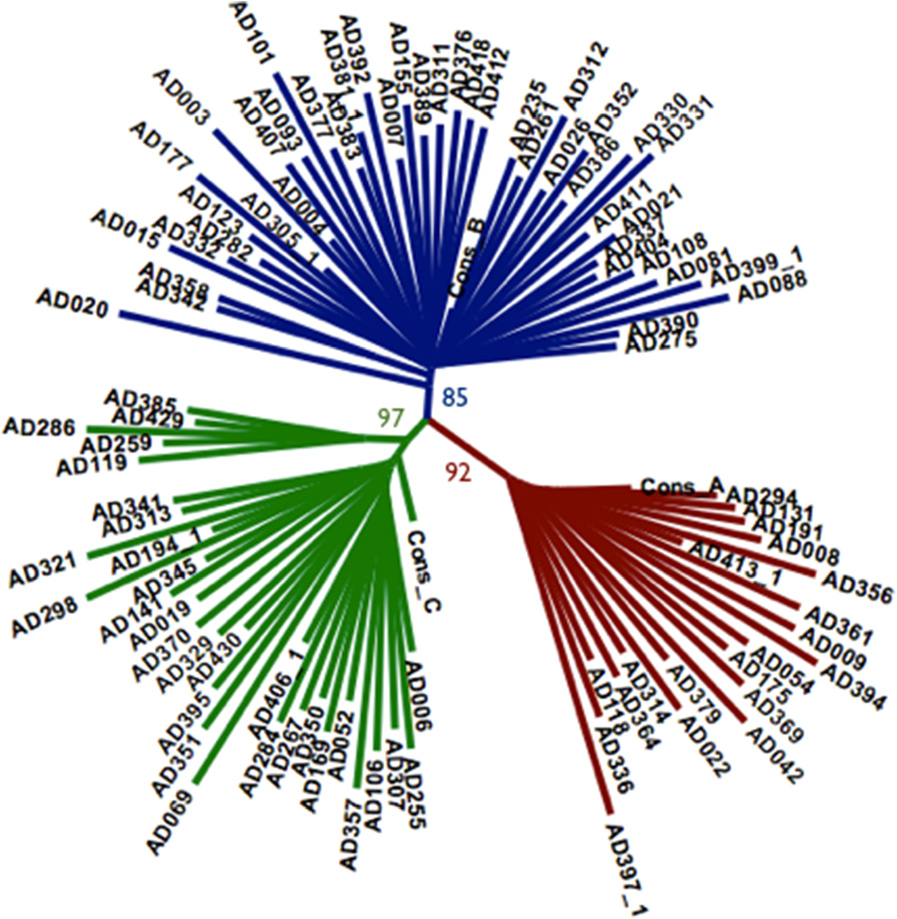
**Unrooted neighbor-joining tree of 91 patient-derived HCV AD78 core-NS2 nucleotide sequences and the three consensus sequences for HCV AD78 variants A, B and C.** Bootstrapping values for each of the three major clades are indicated.

Genomic DNA isolated from EDTA-blood of AD78-infected patients was used for detection of SNPs by real-time PCR (LightSNiP Assay). This test is based on melting curve analysis and allows for detection of mutations located within the sequence segment corresponding to a hybridization probe.

The results have shown that the sequences of most tested HCV cell receptors were very conserved in the anti-D cohort of women of Caucasian origin. The allele frequencies for the most investigated SNPs were below 1% (rs45508991, rs5890, rs74830677, rs77554031, and rs79095497) or 5% (rs11669576). Analysis of the OCLN SNP rs2666626 demonstrated the presence of the G/G allele in DNA sequences from 27 of 91 anti-D patients; the other 64 sequences were positive for the G/C allele. Subsequently, the frequencies of the OCLN SNP rs2666626 genotypes G/G and G/C in patients infected with different HCV AD78 variants were checked (Figure 2). Upon such analysis no significant differences in the frequency of these alleles in subgroups of patients infected with the HCV AD78 variants A, B or C was found, indicating absence of evident correlation between HCV cell receptor polymorphism and presence of a particular virus variant in the anti-D cohort. Thus, the heterogeneity of the major HCV cell receptors does not seem not to play an important role in the mechanism of virus selection at the stage of viral entry in case of natural HCV infection with multiple virus variants.

**Figure 2:**
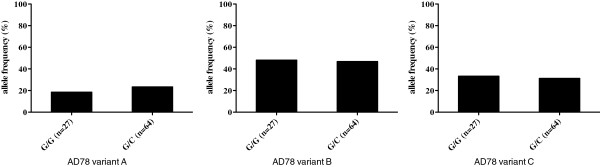
OCLN SNP rs2666626: frequencies of the G/G and G/C allels in patients infected with HCV AD78 variants A, B, and C.

One should note, however, that in the current study heterogeneity of only the major HCV receptor LDLR, SCARB1, and OCLN genes was investigated. In the last few years a number of additional cell factors and co-factors facilitating the HCV entry process have been described [8-10]. These factors include epidermal growth factor receptor (EGFR), ephrin receptor A2 (EphA2), Niemann-Pick C1-Like 1 cholesterol absorption receptor (NPC1L1), and transferrin receptor 1 (TFR1). However, information on the genetic heterogeneity of these proteins at the moment is scarce. That precludes a detailed analysis of a contribution of polymorphism of newly identified HCV entry factors to virus variant selection in case of a complex HCV inoculum.

The fact that anti-D patients did not harbor more than one virus variant might not be attributed only to the HCV receptor heterogeneity. One recent comprehensive study has demonstrated that even minor differences in E1/E2 sequence can result in marked changes of viral fitness [11]. The authors have shown that upon HCV inoculation of SCID/Alb-uPA mice with transplanted human hepatocytes the resulting infections were caused not by virus variants predominant in the inoculum but by variants, which were present there only in minute amounts. One might suggest that in our anti-D cohort the selection of HCV AD78 variants could also be determined on the one hand by differences in their fitness and on the other hand by host genetic factors other than HCV receptors and entry co-factors. It would be of great interest to apply the human liver cells graft-mouse system for studies of infectivity of the HCV AD78 variants present in contaminated anti-D globulin batches and in sera from individual anti-D patients.

## Material and methods

### Patients

A large HCV genotype 1b single source outbreak after immunization of about 3000 women with virus contaminated anti-D immunoglobulin occurred in East Germany in 1978–1979 [5]. Samples from this anti-D cohort were collected since 2008 by members of the East German HCV study group [12]. The HCV RNA–positive samples from 91 of these patients were randomly selected [6] and included in this study.

### DNA amplification and sequencing

Viral RNA from serum samples of 91 infected patients was extracted using the RNeasy kit (Qiagen) and reverse transcribed into cDNA using the Thermoscript kit (Invitrogen) according to manufacture’s instructions. The HCV core-NS2 genomic region (codons 342 to 3419, according to the AF009606 HCV isolate) was amplified in four overlapping fragments by standard PCR techniques. A list of primers is provided as Additional file 1: Table S1. The DNA fragments were gel-purified using the QIAquick Gel Extraction Kit (Qiagen) and cloned into the pCR4-TOPO plasmid (Invitrogen). Sequencing of DNA fragments was outsourced to LGC Genomics in Berlin, Germany. Direct or clonal sequences were subjected to phylogenetic analysis using the Phylip-package (version 3.69) and statistical confidence was estimated from 100 bootstrapped trees.

### SNP analysis

Genomic DNA isolated from EDTA-blood of 91 AD78-infected patients was used for detection of SNPs of the HCV cell receptor genes LDLR, SCARB1, and OCLN by LightSNiP Assay (TibMolbiol) according the manufacturer’s instructions using the LightCycler 2.

## Competing interests

The authors declare that they have no competing financial and non-financial interests.

## Authors’ contributions

SV and MR were the principal investigators, who designed and supervised the study, and wrote the grant application. ML, MW and SV performed the experiments. JT and ML participated in the design of the study and analysis of the results. All authors drafted the manuscript and approved of its final version.

## Supplementary Material

Additional file 1: Table S1 Primers used for amplification of the HCV AD78 core-NS2 genomic region (codons 342 to 3419, according to the AF009606 HCV isolate) in four overlapping fragments. Click here for file
