# Supplementary material for: Genetic heterogeneity of hepatitis C virus cell entry receptors seems to have no influence on selection of virus variants
Source: Virol J. 2014 Mar 14;11:50. doi: 10.1186/1743-422X-11-50 (PMC4007597; doi:10.1186/1743-422X-11-50)
Supplement: Additional file 1: Table S1 — Primers used for amplification of the HCV AD78 core-NS2 genomic region (codons 342 to 3419, according to the AF009606 HCV isolate) in four overlapping fragments. [file 1743-422X-11-50-S1.doc]

**Additional file 1: Table S1 Primers used for amplification of the HCV AD78 core-NS2 genomic region (codons 342 to 3419, according to the AF009606 HCV isolate) in four overlapping fragments.**

| **HCV genome region** | **PCR1** | **PCR2** |
| --- | --- | --- |
| Core | 5’-GAT CAA CCC GCT CAA TGC CTG-3’ (sense)  5’-GCC GCG AGC GTG GGG GTG AGC-3’ (anti-sense) | 5’-TAG CCG AGT AGT GTT GGG TC-3’ (sense)  5’-CCA GCA GCG AGA GGA GTT GTC-3’ (anti-sense) |
| E1 | 5’-TTC GCC GAC CTC ATG GGG TAC-3’ (sense)  5’-AGA ACA GMG CGG CAA KRA ACC-3’ (anti-sense) | 5’-TAT AGA TAT CAT GGG GTA CAT TCC GCT CGT C-3’ (sense)  5’-GTT TTG AGA GAG TCA TTG CAG-3’ (anti-sense) |
| E2-p7 | 5’-AAG CTG TCG RGG ACA TGG TG-3’ (sense)  5’-ATA TGA GCT TAG CGA GGA ACA-3’ (antisense) | 5’-TAT TCC ATG GTG GGG AAC TGG-3’ (sense)  5’-CTT TAT AGT ATG GTG ACA AGG TC-3’ (anti-sense) |
| P7-NS2 | 5’-GGT RTA GGG TCA GTG CTC GTC TC-3’ (sense)  5’-GAC TRG TGA TGA TAC AGC CAA G-3’ (anti-sense) | 5’-TAT CCT RTT GCT CTT CCT TCT C-3’ (sense)  5’-TGA TGA TAC AGC CAA GYA GGC-3’ 8anti-sense) |
